# A Case Report on Iatrogenic Methotrexate Toxicity

**DOI:** 10.7759/cureus.64081

**Published:** 2024-07-08

**Authors:** Kristina L Quan Soon, Sundeep Shah, Ramy Ibrahim, Khalid Alzwahereh

**Affiliations:** 1 Internal Medicine, Premier Medical Associates, The Villages, USA; 2 Research, Premier Medical Associates, The Villages, USA; 3 Medicine, CLS Health, Houston, USA

**Keywords:** immunosuppression, rheumatoid, mucositis, toxicity, methotrexate, leucovorin

## Abstract

This case study studies the adverse effects of methotrexate toxicity as well as the importance of early recognition of the symptoms and signs of methotrexate toxicity. This study involves a 54-year-old female who accidentally took methotrexate in high doses for a period of five consecutively days. The patient had been diagnosed with mixed connective tissue disease and was being treated with methotrexate once weekly. However, she took 7.5 mg (three tablets) three times a day for five days instead of 15 mg once weekly in two divided doses.

The patient immediately went to the ER but was asymptomatic and discharged after a CBC showed values within the normal range. The patient was then seen by her primary care physician and advised to return to the ER. At this time, the patient had mucocutaneous lesions and was complaining of oral and throat pain, and a repeat CBC demonstrated pancytopenia.

The patient was admitted to the hospital for further management and treatment of methotrexate toxicity.

## Introduction

Methotrexate is a medication that inhibits dihydrofolate reductase, an enzyme involved in folate synthesis, which is important for DNA (deoxyribonucleic acid) synthesis and hence cellular proliferation. As a result, methotrexate can have an effect on many different cell types in the human body by inhibiting DNA synthesis. Owing to its non-selective nature, treatment with methotrexate can affect other cells in the body apart from the target tissue/ area. It can affect the bone marrow and gastrointestinal tract resulting in adverse and serious side effects [1,2].

Methotrexate can decrease cellular reproduction and it is often used as an anti-neoplastic drug, an immunosuppressant, and a DMARD (disease-modifying anti-rheumatic drug). It is used for the treatment of certain cancers, autoimmune diseases, ectopic pregnancies, and gestational trophoblastic diseases [3,4].

Methotrexate can be administered in low doses or high doses. Low-dose methotrexate therapy is usually administered orally and is often 7.5 mg to 25 mg weekly or less than 50 mg/m^2^. It is used in the treatment of psoriasis, inflammatory bowel disease, rheumatoid arthritis, mixed connective tissue disease, juvenile psoriatic arthritis, and many other inflammatory conditions [3].

Methotrexate can also be used in high doses. High-dose methotrexate is a dose ≥ 500 mg/m^2^. At high doses, it is usually given intravenously and is used in the treatment of certain cancers such as leukemia, breast cancer, lymphomas, osteosarcomas, and trophoblastic neoplasms. After giving a high dose of methotrexate, patients are usually given a leucovorin rescue to mitigate the side effects of high-dose methotrexate. Leucovorin/folinic acid is reduced folate, which can “rescue” normal cells by providing reduced folate for DNA synthesis and cellular reproduction [4].

While methotrexate’s therapeutic properties often outweigh its toxic effects, methotrexate can have adverse effects that can impact patients’ health and quality of life. The side effects of methotrexate need to be recognized early and managed swiftly to avoid detrimental and irreversible effects on the patient.

The side effects of methotrexate toxicity include stomatitis, abnormal liver chemistries, rash neurotoxicity, nephrotoxicity, gastrointestinal problems, CNS (central nervous system) symptoms, alopecia, drug-related fever, and hematologic abnormalities such as myelosuppression. Gastrointestinal and CNS symptoms are usually seen in the first 24-48 hours of methotrexate toxicity; however, stomatitis and other side effects may take longer and persist for days to weeks [3,4].

This case follows the early recognition of the side effects of methotrexate toxicity as well as the importance of patient awareness of the medications they are prescribed and how they should be used. It highlights the ease with which a medication with many therapeutic uses can have unfavorable outcomes if not used appropriately or as directed.

## Case presentation

This case involves a 54-year-old female patient whose medical history included hypertension, Lyme disease, mixed connective tissue disease, sick sinus syndrome, and ulcerative colitis. She was initially prescribed methotrexate 15 mg once weekly by the rheumatologist for polyarthritis due to her mixed connective tissue disease. However, she erroneously took three (3) 2.5 mg tablets twice a day, a total of 15 mg daily for 5 days consecutively. She then went to the ER (emergency room) complaining of general malaise and feeling unwell and was discharged on antibiotics and diagnosed with folliculitis. Her bloodwork at this time was unremarkable. The following day she presented to the primary care physician with icterus and general malaise as well as significant dysphagia. Upon confirmation of the medication administration error, the patient was sent back to the emergency department for potential leucovorin rescue. The patient returned to the ER department at once, complaining of oral and throat pain, with difficulty swallowing and speaking. The patient also had symptoms of nausea and vomiting. At this time, the patient was admitted to a tertiary care center for higher-level care and treatment. She was diagnosed with methotrexate toxicity.

On examination, the patient was febrile with a measured temperature of 38.5℃, had oral hemorrhagic sores on the hard and soft palate, and had erythematous papules on the buttocks and lower limbs posteriorly.

During the patient’s admission to the hospital, the patient developed diarrhea, hematochezia, and worsening folliculitis A nasogastric tube was placed because the patient’s oral mucositis led to difficulty and pain on swallowing. She also had persistent fevers and experienced extreme fatigue and generalized weakness with difficulty walking. She was also investigated for pneumonitis, but the patient had no signs and symptoms of respiratory distress and the chest radiograph was normal.

Her lab investigations showed worsening anemia, thrombocytopenia, and neutropenia with decreasing absolute neutrophil count (ANC). Her basic metabolic profile (BMP) showed some electrolyte abnormalities, and her renal function was normal as seen in Tables 1, 2. Her serum methotrexate level was <0.04 mg/ml.

**Table 1:** Complete blood count (CBC) results during the patient’s hospital admission at a tertiary care center

Results (Normal Range)	Day 1	Day 2	Day 3	Day 4	Day 5	Day 6	Day 7
WBC (White Blood Cell) K/ul (4.0-10.0)	0.7	0.9	0.5	0.5	0.7	0.9	2.8
Hb (Hemoglobin) g/dL (12.0-16.0)	11	11.2	11.3	9.1	10.2	9.3	9.5
MCV (Mean Corpuscular Volume) fL (80-100)	92.2	95.7	92.7	91.3	91.0	90.8	91.4
Platelet K/uL (150-450)	133	107	77	48	27	15	15
Neutrophil % (40-80)	12	15	6	1	1	13	61
ANC (Absolute Neutrophil Count) k/uL (1.7-7)	0.09	0.14	0.03	0.01	0.01	0.11	1.78

**Table 2:** Basic metabolic profile (BMP) results during the patient’s hospital admission at a tertiary care center

Results (Normal range)	Day 1	Day 2	Day 3	Day 4	Day 5	Day 6	Day 7
Na+ (Sodium) mmol/L (136-145)	137	141	135	137	136	133	136
K+ (Potassium) mmol/L (3.3-5.1)	3.1	3.3	3.4	4.3	3.1	3.9	3.5
Cl- (Chloride) mmol/L (98-107)	106	108	100	118	102	101	104
Creatine mg/dL (0.38-1.02)	0.59	0.57	0.61	0.79	0.49	0.50	0.45
EGFR (Estimated Glomerular Filtration Rate)	>60	>60	>60	>60	>60	>60	>60
Calcium mg/dL (8.4-10.20	8.1	8.2	8.6	7.0	8.1	8.0	8.2

During her admission, a gastroenterologist, hematologist, and wound care doctor were consulted. She was started on intravenous antibiotics, folic acid, granulocyte colony-stimulating factor (filgrastim), and leucovorin. She was also started on an anti-emetic, anti-inflammatory, acetaminophen, and anticoagulant.

**Figure 1:**
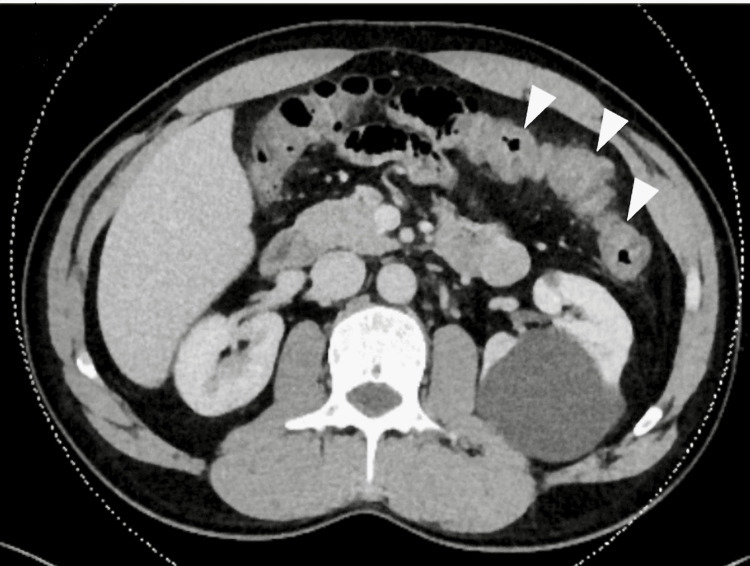
Computed tomography (CT) scan of the abdomen showing colonic inflammation Colitis is indicated by spearhead arrows.

Her medications for ulcerative colitis, mesalamine, and budesonide were stopped A CT scan of the abdomen showed an inflammatory mucositis and colitis (Figure 1), which the gastroenterologist attributed to the methotrexate overdose.

She was also reviewed by the ostomy/wound care doctors for her skin lesions and worsening folliculitis and started on a topical antifungal.

She remained in the tertiary care center for a total of six days, and once stable and showing signs of improvement, she was transferred to the regional general hospital. On discharge, her WBC (white blood cell) count and ANC were improving, she was tolerating light fluids and her weakness had improved slightly. She remained at the general hospital for a further seven days where she was continued on IV antibiotics due to persistent fevers. Filgrastim was also continued for three additional days. Her CBC improved (Table 3), and the patient was well.

**Table 3:** CBC prior to discharge from a regional general hospital

Results (Normal Range)	Day of Discharge
WBC (White Blood Cell) K/uL (4.0-10.0)	7.5
Hb (Hemoglobin) g/dL (12.0-16.0)	9.1
MCV (Mean Corpuscular Volume) fL (80-100)	94
Platelet K/uL (150-450)	299
Neutrophil % (40-80)	58
ANC (Absolute Neutrophil Count) K/uL (1.7-7)	4.4

The BMP showed no abnormalities. The patient was then transferred to a skilled nursing facility for intensive nursing care. The patient’s weakness and mucositis gradually improved, and she was discharged after 14 days.

## Discussion

Effects of methotrexate toxicity

Methotrexate is a very important drug in medicine. It is commonly used to treat hematological, rheumatological, and oncological disorders because of its effect on DNA synthesis and cellular reproduction. However, it is this same effect on cells that contributes to the duality of methotrexate. If methotrexate is not used appropriately, it can have rapid and severe effects [5].

A case report done in 2017 presented two cases in which patients were accidentally prescribed methotrexate instead of digoxin for one and five months respectively, leading to accidental methotrexate poisoning [6]. In a recent cross-sectional study by Ahmadzadeh et al., 8 of the 27 patients studied developed methotrexate toxicity due to incorrect medication consumption, an incorrect dose of medication, and an increase in the frequency of consumption (daily instead of weekly) [7].

Methotrexate toxicity can cause mucocutaneous ulcers and oral mucositis at low and high doses. Mucocutaneous ulcers are an early sign of methotrexate toxicity and can also be a prelude to impending systemic toxicity [6]. In a study done by Kivity et al., 53.5% of patients developed oral mucositis, and a subset of these developed both oral mucositis and cutaneous lesions [8].

The development of gastrointestinal symptoms is also a common side effect of methotrexate use and patients usually develop nausea, vomiting, diarrhea, and loss of appetite. In patients receiving oral methotrexate, there is a higher incidence of diarrhea. Patients are usually given a proton pump Inhibitor or anti-nausea medication, which is usually administered in a cycled weekly manner along with methotrexate administration. However, methotrexate-induced gastrointestinal mucositis can severely affect the whole gastrointestinal tract leading to malabsorption, weight loss, and interruption of medications [2,3].

There are also some cases where methotrexate can cause irreversible or reversible pulmonary toxicity. However, this occurs independent of the dose or duration of methotrexate treatment and can be seen in both high and low-dose treatment [3].

The most common sign of methotrexate toxicity, however, is pancytopenia. Methotrexate is most effective on rapidly proliferating cells such as lymphocytes. In most cases, methotrexate toxicity led to pancytopenia, with neutropenia and thrombocytopenia. Dalkilic et al. reported a 93.5% incidence of pancytopenia/neutropenia with an improvement in approximately five days in their study. The average hospital admission for leukopenia was 5 ± 3.8 days. Similarly, pancytopenia was shown in 82.1 % of patients in a study by Kivity et al., with almost one-third of the patients developing severe neutropenia (neutrophil count less than 500). While the administration of leucovorin and GM-CSF are both important in the treatment of methotrexate toxicity, it is important to note that the outcomes can be unfavorable. Both studies showed that there were patient deaths despite the administration of the treatment due to complications such as infection and bleeding. However, the age and co-morbidities are also confounding or attributing factors in this outcome [8,9].

While the effect of methotrexate varies, it is important to mention that methotrexate can also be nephrotoxic in higher doses and can also affect the renal clearance of the drug, increasing its levels in the plasma. Recent research has shown that patients can develop methotrexate-induced renal failure and that it can have a direct effect on renal tubules and renal tubular enlargement. Pre-existing renal impairment, which often occurs with rheumatological disorder, can also affect methotrexate excretion [2,4].

Lastly, research has shown that despite its extensive and severe side effects, the serum concentrations of methotrexate prove to be inconsequential. Studies have shown that serum levels are often undetectable in patients with pancytopenia and in those with detectable levels of methotrexate, the mean concentration was 0.04 mg/ml [8]. This is also exemplified in a study done by Dalkilic et al., which showed no correlation between serum methotrexate and the severity of symptoms or toxicities [9].

Treatment of methotrexate toxicity

The administration of leucovorin is the mainstay of methotrexate toxicity. It is often administered to reverse the effects of methotrexate by providing a folate supply for cellular activity in normal cells and is important in preventing the gastrointestinal and hematological side effects of methotrexate toxicity. In addition to administering leucovorin, methotrexate must also be stopped immediately [2,3].

In patients with severe febrile neutropenia, a granulocyte-stimulating factor is administered. which stimulates the production of white blood cells by bone marrow and improves the neutrophil count over time. It is often necessary in severe neutropenia. In addition, an IV antibiotic is usually administered to prevent opportunistic infections that can occur due to this acquired immunodeficiency. Blood products, such as platelets and PRBCS (packed red blood cells), can also be administered if patients develop severe thrombocytopenia and anemia [5,7].

Other treatments include hydration with the administration of a suitable diuresis, urine alkalinization with IV administration of sodium bicarbonate, and the administration of glucarpidase. Alkalinizing of the urine makes methotrexate more soluble and facilitates the excretion of methotrexate and its by-products [2,4].

Glucarpidase is usually administered in high-dose methotrexate toxicity, and there must be an elevated serum concentration of methotrexate. It is licensed for the treatment of plasma levels of > 1mmol/L in patients with renal impairment and delayed clearance. It is a recombinant bacterial enzyme that rapidly metabolizes serum methotrexate. Widemann et al. found that glucarpidase decreased methotrexate levels to more than 98% in patients with nephrotoxicity, however, it had no impact on the time to recovery of renal function when compared to other methods. Glucarpidase also metabolizes leucovorin so both cannot be administered simultaneously. It is also very costly and not readily available in even larger hospitals, which further limits its use [1,10].

The treatment of methotrexate varies due to its wide adverse effect profile. It can affect various organs, and each treatment plan needs to be tailored to the patient to be beneficial. It involves a thorough assessment of the patient's past and current history to make an appropriate treatment and management plan because most patients on methotrexate likely have a pre-existing chronic or debilitating condition.

## Conclusions

Methotrexate is a medication whose therapeutic properties often outweigh its adverse effects. However, it is important to recognize its potential to become lethal if not administered appropriately or carefully. It can lead to disastrous outcomes if taken excessively, erroneously, or even chronically if not monitored. Medication reconciliation is an essential part of methotrexate therapy, and the weekly regimen must be explained to the patient, with written confirmation. The rapid onset of symptoms must also be noted when this medication becomes toxic, and medical providers must be vigilant about recognizing the early symptoms of methotrexate toxicity and acting quickly to avoid harmful outcomes.
